# Chronic obstructive pulmonary disease guidelines in Europe: a look into the future

**DOI:** 10.1186/s12931-018-0715-1

**Published:** 2018-01-18

**Authors:** Marc Miravitlles, Nicolas Roche, João Cardoso, David Halpin, Zaurbek Aisanov, Hannu Kankaanranta, Vladimir Kobližek, Paweł Śliwiński, Leif Bjermer, Michael Tamm, Francesco Blasi, Claus F. Vogelmeier

**Affiliations:** 1 Pneumology Department, University Hospital Vall d’Hebron, Ciber de Enfermedades Respiratorias (CIBERES), Barcelona, Spain; 2 Service de Pneumologie et Soins Intensifs Respiratoires, Hôpital Cochin (AP-HP), University Paris Descartes (EA2511), Paris, France; 3 Hospital de Santa Marta, Centro Hospitalar de Lisboa Central, Universidade Nova de Lisboa, Lisbon, Portugal; 4 Royal Devon and Exeter Hospital, Exeter, UK; 5 Pulmonology Department, Pulmonology Research Institute, Russian National Medical University, Moscow, Russia; 6 Department of Respiratory Medicine, Seinäjoki Central Hospital, Seinäjoki, Finland; 7 Faculty of Medicine and Life Sciences, University of Tampere, Tampere, Finland; 8 Department of Pneumology, University Hospital Hradec Kralove, Faculty of Medicine in Hradec Kralove, Charles University in Prague, Hradec Kralove, Czech Republic; 9 2nd Department of Respiratory Medicine, Institute of Tuberculosis and Lung Diseases, Warsaw, Poland; 10 Department of Respiratory Medicine & Allergology, Skåne University Hospital, Lund, Sweden; 11 Clinic of Pulmonary Medicine and Respiratory Cell Research, University Hospital, Basel, Switzerland; 12 Department of Pathophysiology and Transplantation, University of Milan, and Department of Internal Medicine, Respiratory Unit and Adult Cystic Fibrosis Center, Fondazione IRCCS Cà Granda Ospedale Maggiore Policlinico, Milan, Italy; 13 Department of Medicine, Pulmonary and Critical Care Medicine, University Medical Center Giessen and Marburg, Philipps-University Marburg, Member of the German Center for Lung Research (DZL), Marburg, Germany

**Keywords:** Chronic obstructive pulmonary disease, Clinical practice guidelines, Treatment recommendations

## Abstract

Clinical practice guidelines are ubiquitous and are developed to provide recommendations for the management of many diseases, including chronic obstructive pulmonary disease. The development of these guidelines is burdensome, demanding a significant investment of time and money. In Europe, the majority of countries develop their own national guidelines, despite the potential for overlap or duplication of effort. A concerted effort and consolidation of resources between countries may alleviate the resource-intensity of maintaining individual national guidelines. Despite significant resource investment into the development and maintenance of clinical practice guidelines, their implementation is suboptimal. Effective strategies of guideline dissemination must be given more consideration, to ensure adequate implementation and improved patient care management in the future.

## Background

The ultimate treatment goals in chronic obstructive pulmonary disease (COPD) management remain uniform across the majority of national and international COPD clinical practice guidelines (CPGs), and include reduced symptoms, reduced exacerbation risk and improved quality of life [1]. To achieve these goals, CPGs require regular updates with recent and relevant state-of-the-art medical and scientific developments. Guidelines strive to improve the quality of healthcare and to reduce variations in the treatment and management of COPD [2].

Clinical practice guidelines provide recommendations on patient management based on available evidence and, in certain cases, educated opinion where there is no direct evidence available [3]. The quality of the available evidence and the intended audience of CPGs remain core considerations for their development [4]. In recent years, CPGs have further evolved in response to an increasing recognition of the need for more stringent, systematic approaches when recommending specific therapeutic interventions or strategies [5]. The importance of rigorous processes to ensure that only accurate and appropriate treatment recommendations are made is now well-accepted among professional scientific societies. In fact, standards to guide the preparation of CPGs are now available [6]. However, little attention is afforded to the challenges and pitfalls associated with the development of such documents.

A concerted effort between multiple stakeholders is needed to ensure precise, practical and up-to-date clinical recommendations for the diagnosis and optimal management of COPD. Guidelines must be locally relevant; therefore local expert stakeholders should offer local proposals, while referring to global evidence-based documents.

This review article highlights the challenges associated with the development and implementation of national CPGs for COPD in Europe and Russia.

### Current challenges associated with the development of guidelines

Important advances in the methodologies used for the development of CPGs have been made in recent years. These include the Grading of Recommendations Assessment, Development and Evaluation (GRADE) guides [7]. GRADE provides a transparent, systematic approach to review available evidence and rate its quality, in order to make recommendations of graded strength based on the degree of confidence in the benefit-risk-cost ratio and applicability of the strategies of interest. GRADE is best used in response to defined key clinical (population/intervention/comparator/outcome [PICO]) questions [5, 8, 9].

Evidence-based CPGs should require minimal interpretation by end-users, to reduce the risk of bias. However, this type of CPG also carries inherent drawbacks and limitations, including a potential disconnection between the focus on high-quality scientific evidence and real-world clinical practice [10]. Evidence-based guidelines may not address areas where there is an insufficient number of well-designed clinical trials (i.e. evidence), but yet clinicians still require guidance in these areas [11].

While rigorous methodologies such as the GRADE strategy offer robust and less biased treatment recommendations, solely relying on such strict methodologies can also compromise the conclusions and external validity of CPGs. Using high-level, formal methodology to develop guidelines may exclude clinically relevant study results [12]. Evidence-based guidelines may reduce professional autonomy or clinical judgement [11]. Furthermore, the evidence included in these grading processes is primarily collected from specific subsets of patients who meet strict inclusion criteria for participation in large clinical trials: specifically, registration randomised controlled trials (RCTs) recruit patients in whom there is the highest chance to demonstrate the efficacy of tested agents [10]. Consequently, often the results obtained do not allow determination of (i) the generalisability of results obtained in selected populations, or (ii) the most suitable target subgroups in terms of benefit-risk ratio. However, it is well-recognised that significant heterogeneity exists among patients with COPD. Older or very severe COPD patients, patients with a history of asthma and/or allergy, never smokers or patients with multiple comorbidities may not meet inclusion criteria and therefore may not be represented by the evidence used to support most recommendations within guidelines [3, 11]. A recent study by Halpin et al. reported that only 27% of COPD patients are eligible for RCTs [13]. Under-representation of “challenging patients” (i.e. those who do not meet strict eligibility criteria, particularly the elderly, those with significant comorbidities and those requiring long-term oxygen therapy) in RCTs results in evidence-based CPGs that may not be fully adequate to ensure the optimal management of a significant number of patients.

Conversely however, guidelines which rely heavily on consensus opinion, rather than high-quality evidence, may be vulnerable to bias and individual interpretation [11]. To try to find the right balance, evidence from real-world effectiveness studies should be more heavily considered in CPGs. These could include the results of observational studies or pragmatic trials where appropriate [10], provided that they satisfy appropriate quality standards [14]. In light of this, CPGs should ideally combine both evidence- and opinion-based approaches in a complementary and transparent way. This can be achieved by clearly highlighting sections that are evidence-based, and addressing gaps in the knowledge by educated opinion or extrapolation from efficacy evidence in other disease areas. Furthermore, of significant consideration is the use of single-disease guidelines for patients with multiple comorbidities. This may be particularly relevant in the case of COPD, because due to the advanced age of the majority of patients and the exposure to noxious particles or gases, especially tobacco smoking, the prevalence of comorbidities is substantial [15]. In addition, COPD and its respiratory consequences can exert direct deleterious effects on other systems; one example is the effect of lung hyperinflation on heart function [16]. Conversely, some comorbidities can increase the burden of COPD; e.g., anxiety and depression can increase the perception of dyspnea through various mechanisms including hyperventilation and psychological distress [17]. The frequency and type of comorbidities presented may be different in patients in real life compared to those included in RCTs [18]; therefore, guidance on multimorbidity will need to be considered in the future [19–21].

Guidelines are time-consuming and expensive to produce. Extensive literature reviews and detailed analyses require more time and resources than clinical and academic experts can dedicate to the development and updates of national or international CPGs [12, 22–24]. This puts strain on local and national societies with limited funding. Budget and available resources are important factors for most countries when developing and updating their national guidelines [22]. Financial support from private (e.g. pharmaceutical) companies may raise issues around potential conflicts of interest [24]. New simplified strategies for CPG development are being tested; they combine consensus through a Delphi methodology with strict application of GRADE in areas where consensus is not reached or that are subject to a high risk of bias [25]. If adequately validated, these strategies could save significant time and resources. An overview of the developmental processes for national CPGs in Europe and Russia is presented in Table 1.

**Table 1:** The development of national guidelines in Europe and Russia: participants and intended audiences

Country	Evidence system used	Organisation involved in the development	Participants involved in the development	Intended audience	Reference
Czech Republic	Consensus	The Czech Pneumological and Phthisiological Society (CPPS) commissioned an expert group to draft recommended guidelines for the management of stable COPD. Subsequent revisions were further discussed at the National Consensus Conference. Reviewers’ comments contributed to the establishment of the final version.	Pulmonologists and pharmacologists	Pulmonologists (full version), internists, GPs, and emergency physicians (reduced version). The Czech national recommendation was fully accepted by the State Institute for Drug Control (SUKL).	[43]
England	NICE technical manual methodology (includes GRADE)	National Institute for Health and Care Excellence (NICE)	Pulmonologists, GPs, respiratory nurses, physiotherapists patients, NICE technical team (including health economists), feedback from registered stakeholders (payors, professional bodies, hospitals etc.)	Pulmonologists, GPs, other specialists, all other healthcare professionals involved in caring for people with COPD, payors and managers	https://www.nice.org.uk/process/pmg20/chapter/introduction-and-overview [44]
Finland	Evidence-based medicine and GRADE methodology	The Current Care Guidelines were developed by the Finnish Medical Society Duodecim in association with various medical specialist societies. The guidelines were produced with public funding and are open to all healthcare professionals and the general public, and include patient versions. A large reviewing group including GPs was asked to comment on the guideline.	Pulmonologists, GPs and internists	Pulmonologists, GPs, other specialists, all other healthcare professionals (including nurses, physiotherapists, pharmacists) and citizens	www.kaypahoito.fi/web/english/home [41, 45]
France	(A) Position paper/statement on pharmacological treatment optimisation of stable COPD	A restricted expert group was commissioned by the national society (SPLF) to produce an initial proposition. A larger reviewing group including GPs was asked to comment.	Pulmonologists and GPs	Pulmonologists and GPs	[46]
	(B) GRADE method for guidelines on exacerbations	An extensive multidisciplinary group of experts and end-users was commissioned to produce the initial document, which was commented on by a panel of external reviewers.	Pulmonologists, GPs, intensivists, emergency physicians, physiotherapists and nurses	Pulmonologists, GPs, intensivists, emergency physicians, physiotherapists and nurses	[47]
Germany	Consensus	The German Respiratory Society (DGP) and the German Airway League (AWL) commissioned an expert group to develop a guideline for the diagnosis, assessment and management of COPD.	Pulmonologists	Pulmonologists, GPs, intensivists, emergency physicians, physiotherapists, nurses and patients	Vogelmeier CF et al. Pneumologie 2017; in preparation
Italy	Consensus	The document was prepared by a working group appointed by the three major national respiratory societies (AIMAR, AIPO and SIMeR) and the Italian Society of General Medicine (SIMG). Representatives of the Italian Ministry of Health and AGE.NA.S. were involved as external independent observers to ensure ethical, social and solidarity principles.	Pulmonologists and GPs	Pulmonologists and other specialists working either inside or outside the hospital setting, GPs, other healthcare professionals, patient associations, and institutions at national, regional, or local level	[48]
Poland	Consensus	Polish Respiratory Society	Pulmonologists	Pulmonologists and a short version for GPs	[49]
Portugal	Consensus	National Health Authority (DGS) commissioned an expert group, including National Physicians Organization (OM) and Portuguese Respiratory Society (SPP) representatives, to produce a guidance document.	Pulmonologists	All Physicians of the National Health Service (SNS)	[50]
Russia	Evidence-based medicine and consensus	Russian Respiratory Society and Russian Ministry of Health.	Pulmonologists	Pulmonologists, GPs, other healthcare professionals, patient associations, and institutions at national, regional, or local level	[51]
Spain	GRADE and consensus	11 medical scientific societies and the National Association of Patients.	Pulmonologists, GPs, internal medicine, rehabilitators, nurses, physiotherapists, geriatricians, emergency specialists and patients	Pulmonologists, GPs, internal medicine and emergency specialists	[52]
Sweden	(A) Evidence-based medicine and consensus	Swedish Medical Products Agency (MPA)	Pulmonologists, GPs, allergologists and physiotherapists	Pulmonary specialists, GPs and internal medicine specialists	https://lakemedelsverket.se/upload/halso-och-sjukvard/behandlingsrekommendationer/Kroniskt_obstruktiv_lungsjukdom_KOL_behandlingsrekommendation.pdf [53]
	(B) Evidence-based medicine and GRADE	The Swedish National Board of Health and Welfare	Pulmonologists, GPs, allergologists and physiotherapists	Pulmonary specialists, GPs and internal medicine specialists	[54]
Switzerland	Consensus	The Swiss Society of Pneumology (SGP) commissioned an expert group including pulmonologists from all five University Hospitals in Switzerland, a representative from a REHAB Clinic and at least one representative from each Language Region in Switzerland	Swiss medical doctors (members of the Foederatio Medicorum Helveticorum [FMH])	Pulmonologists, GPs, internal medicine and specialists	[55]

*Abbreviations*: *GP* general practitioner, *GRADE* grading of recommendations assessment, development and evaluation

### The key target audiences of national and international guidelines

Identifying the key target audience is a critical step in the development of CPGs. The audience is broad, and includes healthcare practitioners with varying levels of specialisation and expertise, as well as non-healthcare professionals [26]. Pulmonologists, general practitioners (GPs), other healthcare professionals, patients, payors and policy-makers are the primary audiences of COPD guidelines.

Although not directly involved in the delivery of patient care, healthcare payors, policy-makers and regulatory agencies also comprise the readership of CPGs. Recommendations that relieve the burden of disease (e.g. reduce the frequency or severity of costly exacerbations) or strategies to encourage early diagnosis in COPD patients can be of considerable benefit to payors. Payors rely on CPGs and robust efficacy evidence in order to make informed decisions on funding and reimbursement policies of specific therapies [27, 28]. Policy-makers also need CPGs to develop adequate prevention strategies and to build pathways of care [29]. Guidelines are heavily regarded by regulatory authorities, which may impact the design of clinical trials. This is of significant importance when regulatory authorities adopt the definition or diagnostic criteria used by a particular guideline when defining the requirements for novel drugs, thereby (and perhaps inadvertently) influencing the design of clinical trials [30].

### Who is involved in the development of CPGs?

Guidelines that are intended for widespread use in clinical practice should include relevant stakeholders at various and appropriate stages of the development process, which may encourage improved implementation and adherence of the recommendations through an increased sense of ownership [31, 32]. Although it may not be appropriate for all stakeholders to take an active role in the development process from the beginning, certain groups may participate in the drafting of recommendations or at the review stages.

Academic or clinical expert involvement in guideline preparation should include GPs, pulmonologists, nurses and physiotherapists where appropriate [31]. As the majority of COPD care is administered by GPs, their involvement in guideline development may drive increased primary care physician-specialist communication and integration, which is crucial in the management of COPD, particularly when patient referral is necessary. Input from nurses, cardiologists, physiotherapists and dieticians may also add value and clinical expertise to guidelines in the pathways of care [26].

Importantly, guidelines should involve all “end-users”, including patients, non-expert practitioners and payors to ensure that the guidelines address the right questions from a family or society perspective. Critically, this may also help to incorporate patient preferences into the guidelines. Increased involvement may encourage patients to play a more active role in their healthcare management [33].

### A standardised pan-European guideline; is this realistic?

Duplicate efforts are made across Europe, with individual countries investing significant resources into the development of CPGs [34]. Institutional collaboration and consolidation of efforts may significantly reduce the cumbersome nature of guideline development and frequent updates [35]. Furthermore, variations in individual sets of guidelines will inevitably continue unless collaboration is encouraged and optimised between countries. These variations, however minor, have the potential to mislead or confuse practicing healthcare professionals [10].

Most national guidelines in Europe have been influenced to varying extents by GOLD. Moreover, GOLD 2017 [36] will likely impact future revisions of European national guidelines in a move towards more personalised treatment of COPD. The GOLD strategy also carries a major positive advantage in that the document is updated annually with the most recent and relevant literature and studies; however, no formal evaluation of evidence (i.e. GRADE or similar) is performed. In addition, since by its definition, GOLD aims to provide a global strategy document, some recommendations may not be directly (i.e. without any adaptation) applicable in some areas or contexts. As most countries do not have the resources to facilitate an annual update to their national guidelines, each country has the opportunity to adopt specific sections of GOLD that are locally relevant. Such processes could be facilitated by tighter collaboration between GOLD committees and regional, national or local initiatives.

Owing to the importance of national guidelines, coupled with the international availability of the GOLD 2017 document, there may be potential for the development of an intermediary document between the two. This could comprise a single detailed “umbrella” evidence base supporting common principles but with an adaptation of recommendations to reflect local practices. Individual sensitivities could therefore be facilitated within this common adaptable template. National reimbursement policies, availability of resources and/or regulatory legislation may cause guideline recommendation and prescription deviations between countries. In brief, first-line treatment recommendations (at the class level) and secondary recommendations could be included within the common guideline, with local alternative suggestions added at a local level in line with local policy and scientific societies.

To support the introduction and implementation of a common, adaptable European guideline, a pan-European guideline development resource repository could be compiled as a support tool. Moreover, different sections of a guideline dedicated to a specific healthcare practitioner role may boost implementation across clinical practice.

There is potential for significant alleviation of time and budget constraints through a concerted, collaborative effort between European countries. Who should take the lead during such a collaboration remains to be discussed, but it is likely that the European Respiratory Society (ERS) is in the best position to lead such a project. The ERS is committed to the development of high quality CPGs [37], and either alone or in concert with sister societies such as the American Thoracic Society (ATS), has also delivered evidence-based CPGs for the management of COPD [38, 39]. Developing a European collaboration would be best achieved through the ERS and national societies, agreeing on a common methodology. Tight links with the GOLD group could also be useful to share retrieved evidence and increase reactivity, allowing a continuous update and adaptation process.

### Can COPD guidelines be simplified?

There is an apparent contradiction between the exponential increase in the scientific knowledge of COPD complexity (phenotypes, endotypes, comorbidities etc.) and the need for simplified treatment pathways. Complex raw data needs to be aggregated and translated into meaningful, useful information to support recommendations of new treatments [29]. Algorithms may be helpful to guide COPD therapy in a simple, stepwise and coordinated manner [40]. Such algorithms need to be flexible and continuously evolving in order to remain up-to-date and clinically relevant. Importantly, the availability of algorithms does not negate the need for scientific principles, and the role of clinical judgement should always be acknowledged.

### Considerations for effective guideline dissemination

Once finalised, the CPGs should be shared in many ways to ensure optimal dissemination. Freely accessible online publishing of the guidelines is important. A way forward could be to amalgamate CPGs on all diseases into one single portal that is accessible by all physicians, other healthcare professionals and the general public free of charge. This has been done in Finland by the general Medical Society Duodecim Current Care Guideline system where guidelines on more than 100 diseases are collated on a single online portal and used by most healthcare professionals [41]. Also, presenting the guidelines at local, national and international congresses may increase awareness amongst a myriad of healthcare practitioners. Furthermore, innovative methods to inform relevant end-users of CPGs could be considered e.g. e-mail blasts or social media communications. Plain language summaries may also prove helpful to guide patients and their relatives on available treatments. A short pocket version should be made available to all physicians to facilitate quick and easy access during patient consultations. Useful treatment algorithms should be available on an easily-navigable website. Using smart technology may also improve the implementation of guidelines. Such applications may also have a place within already-existing clinical integrated management systems such as GP practice computer software.

The final presentation of the recommendations should also be carefully considered. Succinct and concise recommendations presented in an easily-digestible format such as tables or charts should be considered for busy healthcare practitioners [10].

## Conclusions

Evidence-based CPGs are rigorous by their very nature, but are difficult to implement in real-life clinical practice [11]. Some suggestions for improvement in the development and implementation of COPD CPGs are presented in Table 2. The authors suggest that an ideal COPD guideline document should comprise a fair balance between evidence-based and expert opinion-based recommendations where definitive evidence is unavailable. For transparency, each recommendation should clearly state whether it is supported by evidence or based on expert opinion and clinical judgement.

**Table 2:** Suggestions for improvement in the development and implementation of COPD guidelines

Development of CPG must be based on a validate method of evaluation and grading of evidence (GRADE or similar)	
In areas in which GRADE may not be applied, consensus or opinion-based recommendations should be incorporated	
A clear identification of evidence-based and consensus-based recommendations is mandatory for transparency	
Clear and simple algorithms are necessary for interpretation and implementation	
All stakeholders must participate in CPGs development	
A common European guideline could be used as a reference and can be adapted to local health systems	
The European Respiratory Society could be the platform to generate and discuss national European CPGs	
Dissemination and adherence is crucial and new technologies may help to this objective	
New studies are required to evaluate the impact of CPGs on clinical and economic outcomes in COPD	

The key target medical audience of COPD guidelines include pulmonologists and GPs, with patients, payors and policy-makers also comprising the intended audience. All end-users should be involved in the development of guidelines. Formally assessing what they expect from the guidelines and which barriers may impede their implementation could help overcome current insufficiencies in routine care for COPD patients.

Some guideline developers struggle to provide the necessary resources to support the development and/or regular update of their guidelines. Furthermore, it is possible that not all aspects of international guidelines will be directly relevant to all local patients. In this regard, improved collaboration between European countries and the ERS may be beneficial, where a single intermediary strategy document for the management of COPD should be considered. This document could encompass some overarching high-level common principles, with the important opportunity for local adaptation. This may significantly reduce the costs and resources associated with guideline development for many countries.

Although the majority of COPD management is conducted by GPs, familiarity of guidelines amongst general practitioners is suboptimal. Approximately 24% of general practitioners have reported that they are not familiar with the GOLD strategy for COPD [42].

Quick and easy access to guidelines on a website or smart phone is important to maximise guideline implementation. A quickly- and easily-understandable treatment algorithm may help to simplify COPD guidelines. The algorithm must be flexible and continually evolving in accordance with new research and evidence.

There is still a paucity of knowledge on the effect of adequate COPD guideline implementation on disease management and patient outcomes. Further studies are warranted to address this gap in the literature.

## Abbreviations

ATS: American Thoracic Society
COPD: Chronic obstructive pulmonary disease
CPG: Clinical practice guidelines
ERS: European Respiratory Society
GOLD: Global Initiative for Chronic Obstructive Lung Disease
GP: General practitioner
GRADE: Grading of Recommendations Assessment, Development and Evaluation
PICO: Population/intervention/comparator/outcome question
RCT: Randomised controlled trial
